# Photocatalytic, Bactericidal and Molecular Docking Analysis of Annealed Tin Oxide Nanostructures

**DOI:** 10.1186/s11671-021-03495-1

**Published:** 2021-02-10

**Authors:** Muhammad Shahid Sharif, Muhammad Aqeel, Ali Haider, Sadia Naz, Muhammad Ikram, Anwar Ul-Hamid, Junaid Haider, Irfan Aslam, Asma Nazir, Alvina Rafiq Butt

**Affiliations:** 1 Physics Department, Lahore Garrison University, Lahore, 54000 Pakistan; 2 Solar Cell Applications Research Lab, Department of Physics, Government College University Lahore, Lahore, 54000 Punjab Pakistan; 3 Department of Clinical Medicine and Surgery, University of Veterinary and Animal Sciences, Lahore, 54000 Punjab Pakistan; 4 Tianjin Institute of Industrial Biotechnology, Chinese Academy of Sciences, Tianjin, 300308 China; 5 Center for Engineering Research, Research Institute, King Fahd University of Petroleum and Minerals, Dhahran, 31261 Saudi Arabia; 6 Physics Department, University of Sialkot, Sialkot, 51040 Pakistan

**Keywords:** Nanoparticles, Annealing, XRD, HR-TEM, Antimicrobial, Dye degradation

## Abstract

Nanosized tin oxide was fabricated with a simple and cost-effective precipitation technique and was analyzed by performing x-ray powder diffraction (XRD), Fourier-transform infrared (FT-IR) spectroscopy, high-resolution transmission electron (HR-TEM) microscopy, energy-dispersive x-ray (EDX) and UV–Vis spectroscopy. The XRD results revealed that tin oxide particles possessed typical orthorhombic structure and exhibited improved crystallinity with annealing. Calcination at 250 °C produced predominantly orthorhombic SnO which transformed to SnO_2_ at higher temperatures of 500 and 750 °C. HRTEM and FESEM images showed existence of agglomeration within the particles of tin oxide. The absorption was found to increase up to a certain annealing temperature followed by a decrease, which was recorded via UV–Vis spectroscopy. The effect of annealing temperature on dye decomposition behavior of synthesized photocatalysts was studied. It was noted that annealing temperature affects the size of synthesized particles, band gap width and photoactivity of tin oxide. The sample prepared at 500 °C followed first-order kinetics and exhibited maximum photocatalytic reactivity toward methylene blue. The experimental results obtained from the present study indicate that SnO_2_ is a promising and beneficial catalyst to remove contaminants from wastewater and environment. The antimicrobial evaluation of SnO annealed at 500 °C against selected targets such as *E. coli* and *S. aureus* depicted significant inhibition zones in comparison with 250 and 750 °C samples. Furthermore, molecular docking predictions of SnO_2_ nanoparticles (NPs) were performed against active pocket of *β*-lactamase and DNA gyrase enzyme belonging to cell wall and nucleic acid biosynthetic pathway, respectively. The fabricated NPs showed good binding score against *β*-lactamase of both *E. coli* (− 5.71 kcal/mol) and *S. aureus* (− 11.83 kcal/mol) alongside DNA gyrase (− 9.57 kcal/mol; *E. coli* and − 8.61 kcal/mol; *S. aureus*). These in silico predictions suggested SnO_2_ NPs as potential inhibitors for selected protein targets and will facilitate to have a clear understanding of their mechanism of action that may contribute toward new antibiotics discovery.

## Introduction

Exceptional properties and a wide ranging technological applications associated with conventional metal oxides provided the impetus to explore these materials in their nanostructured form. Among these, tin oxide (SnO_2_) is considered to be an important metal oxide [1] that exhibits 3.6 eV wide band gap at room temperature [2]. It is n-type semiconducting material that has intrinsic defects in the form of oxygen vacancies with interstitial tin atoms that interact with donors (n-type carriers). An increase in the number of free electrons in conduction band results in an increase in the conductivity of material [3].

The use of SnO_2_ as an oxidation catalyst, photocatalyst, gas sensor and transparent conductor presents the basis to investigate this material further [4–9]. It has the ability to detect flammable, explosive and toxic gases [10]. Industrialization has led to an increase in the discharge of harmful air and water pollutants such as CO and SO_2_ into the atmosphere and harmful azo dyes in wastewater. It has been estimated that approximately 500 tons of various dyes are discharged into industrial wastewater and a major portion (~ 80%) of them is from textile industry [11]. Chemical toxins and organic fuels form part of air pollution while dangerous dyes from water bodies affect earth’s ecosystems, thereby increasing the importance of technology used to detect and prevent such pollutants from harming the environment. Due to its unique physicochemical properties, SnO_2_ has the ability to operate at low temperatures. Due to its inherent non-stoichiometry, it reduces harmful gases by allowing easy adsorption of oxygen on its surface. Moreover, it entails lower cost when compared to other available materials used for similar applications. It also possesses substantial optical transparency and electrical conductivity rendering it suitable for use in optoelectronic components [12]. It is employed in the manufacturing of transparent electrodes and solar cells for use in panels and several electro-chromic devices [13–17].

To prevent waterborne diseases, the removal of bacteria from wastewater is important for drinking and sanitation systems. Between 2003 and 2005 in the USA, four waterborne diseases were reported, which were manifested to pathogens in drinking water affecting about 282 humans. Conventional techniques for disinfection of water bodies are dependent upon chemical agents that are less effective against cyst-forming protozoa (Giardiaand Cryptosporidium). Also, sometimes these techniques produce harmful by-products. Nanotechnology is a new generation technology that can influence world economy via new consumer products, materials usage and manufacturing methods [18]. Metal oxide nanostructures depict enhanced antimicrobial properties attributed to their high surface to volume ratio, stability and biocompatibility. They have unique ability to penetrate through cell membrane structure and destroy cellular parts of bacteria [19].

Two basic tin oxides are mostly studied such as SnO and SnO_2_, and these oxides existence is attributed to dual Sn valance degrees (with oxidation) + 2 and + 4. These two oxides are also called wide band gap semiconducting materials with PbO structure for tin oxide and tetragonal lattice (rutile structure) for SnO_2_ [20]. It possesses wide band gap energy from 3.6 to 4.0 eV, n-type semiconducting material and more than 85% transparency [21]. SnO is a p-type material with band gap ranged from 2.7 to 3.4 eV but experimentally attained band gap may be reached to 3.6 eV. Furthermore, structural, optical and electronic properties of tin oxide indicate that increment in pressure leads to nonstructural orthorhombic formation of SnO and transmittance in UV–visible and near-infrared regions. The increase in temperature also results orthorhombic SnO structure and tetragonal SnO_2_ formation. So, increase in pressure or temperature shifts absorption edge and increases band gap energies. The fabricated polycrystalline SnO can be converted into SnO_2_ phase by increasing temperature from 400 to 700 °C [22]. Due to tuning in band gap tin oxides have been used in electronics industry.

SnO and SnO_2_ materials have also been used in Li-ion batteries [23–25]. The reported literature demonstrates that electrochemical performance of nanomaterials can be improved by controlling its size [26]. Kida et al. reported that a decrease in particle size caused an increase in response of the sensor for H_2_ detection; however, the response to H_2_S and CO increased with increasing particle size [27]. Various methods to produce SnO_2_ have been reported in the literature. Merlin [28] synthesized its nanoparticles with a size range of 20–30 nm via green synthesis using ethanolic-stevia rebaudiana plant extract that acted as capping and reducing agent. Janardhan et al. [29] prepared SnO nanoparticles with an average size of 50 nm by using dilute HCl and SnCl_2_·2H_2_O. Selvakumari et al. [30] fabricated SnO_2_ particles with an average crystal size of 13–40 nm by using chicken eggshell membrane.

Generally, nanomaterials can be synthesized by employing various methods including electrochemical reduction [31], sol–gel [32], hydrothermal [33] and co-precipitation [34]. In the present work, the precipitation method was preferred since it constitutes a convenient and cost-effective technique to synthesize nanostructures. The aim of this study was to observe the effect of various temperatures (250, 500 and 750 °C) used during synthesis on crystallite size, morphology and band gap energy of prepared nonstructural materials. Further, the photocatalytic behavior of synthesized product was studied by employing it for degradation of methylene blue dye that is a commonly encountered pollutant in industry worldwide. In silico molecular docking predictions were performed to unveil mechanism involved in bactericidal activity of SnO_2_ against *β-*lactamase belonging to cell wall biosynthetic pathway and DNA gyrase of nucleic acid biosynthetic pathway from *E. coli* and *S. aureus*.

## Methods

The current study was aimed to synthesize SnO_2_ nanoparticles by a facile and simple precipitation process and annealed at various temperatures. Annealed SnO_2_ was used to remove organic pollutants from wastewater and antibacterial potential.

### Chemicals

**Fig. 1:**
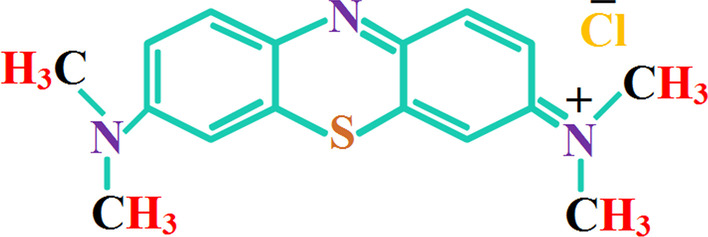
Chemical structure of MB contaminant

Tin(II) chloride dihydrate (SnCl_2_·2H_2_O) and ethanol of analytical grade were acquired directly from Sigma-Aldrich (Germany). Sodium hydroxide (NaOH) and methylene blue (MB) were procured from Merk (Germany) and BDH (UK), respectively. Distilled water was purchased from local market to fabricate tin oxide nanostructures. Chemical structure of the pollutant (i.e., MB dye) used in this study is depicted in Fig. 1.

### Preparation of Tin Oxide Nanostructures

**Fig. 2:**
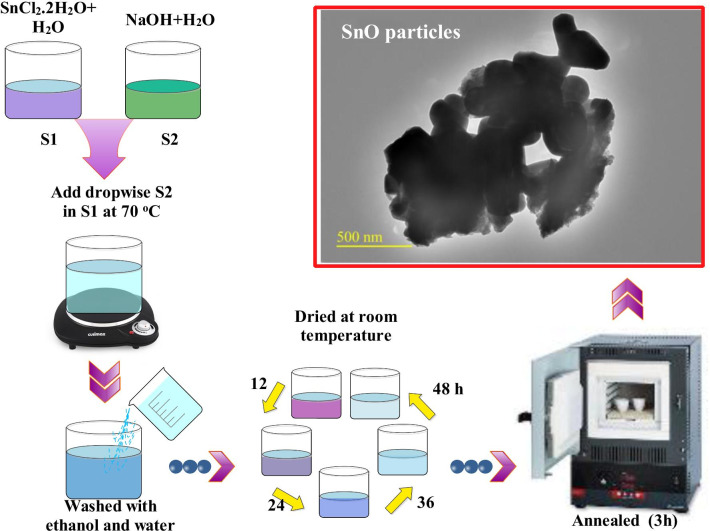
Schematic representation of synthesis of tin oxide samples

In this study, commercial chemicals of analytical grade with high purity were used to prepare tin oxide nanomaterial using precipitation procedure (see Fig. 2). Aqueous solutions of SnCl_2_·2H_2_O (19 g) and NaOH (8 g) were prepared in 50 mL distilled water separately. Aqueous solution of NaOH was poured dropwise in SnCl_2_.2H_2_O solution (~ 10 drops/min) under constant stirring at 70 °C using 400-mL flask. White precipitates appeared upon dropwise addition of solution. The attained product was washed several times using distilled water and ethanol with centrifuge machine. Obtained material was further dried at room temperature for 48 h to remove water content. Finally, dried solid product was ground into fine powder form using mortar and pestle. Three samples were annealed in muffle furnace at various temperatures (250, 500 and 750 °C) for three hours with heating rate 0.5 °C/min [35].

### Photocatalytic Activity Process

**Fig. 3:**
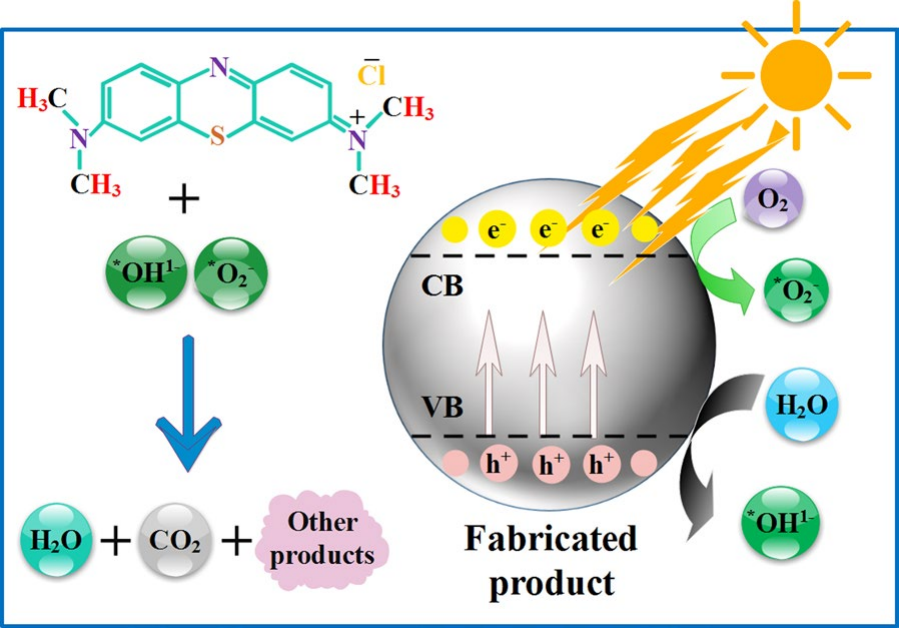
Illustration of degradation of methylene blue MB by photocatalyst

The photoactivity potential of annealed tin oxide samples was evaluated by monitoring photodegradation of MB aqueous solution under light source; mechanism illustrated in Fig. 3 [36–38]. For each photoactivity experiment, known mass of nanomaterial (10 mg) was added into 60 mL aqueous solution of dye (10 mg/L). Prior to light exposure, suspension was stirred magnetically in dark for 5 min [38] to obtain adsorption/desorption equilibrium of dye on the surface of photocatalyst. The suspension solution was irradiated for 80 min with a mercury lamp (400 W and *λ* = 400–700 nm) under stirring (220 rpm). MB samples of ~ 5 mL were drawn out from test solution to monitor MB residual quantity using UV–Vis spectrophotometer ranged 300–750 nm. Variation in MB maxima absorption wavelength (~ 665 nm) to radiation time was recorded to study photodegradation of MB dye. The activities of synthesized products were evaluated by calculating dye degradation % using the following relation:

1 $$\hbox{Degradation}\, \% = [{({C}}_{0}-{C})/{{C}}_{0}]\times 100$$

where *C*_0_ denotes initial MB concentration and *C* presents concentration after irradiation. Without photocatalyst, control experiment was also performed under the same conditions. To examine reusability of nanomaterials, degraded MB solution was centrifuged (6000 rpm/min) for 10 min to recover photocatalyst. Later, recovered product was dried at 80 °C (2 h) to reuse it for MB degradation experiments. To test durability of the catalyst, recycling photoactivity experiments were performed up to four cycles. After each cycle, product was centrifuged, dried and used for next photo-experiment.

### Antimicrobial Activity

In vitro antibacterial action of fabricated SnO at various temperature treatments was valued against most prevalent pathogenic bacteria *E. coli* and *S. aureus* isolated from caprine mastitis using well diffusion assay. Petri dishes containing bacteria activated growth (0.5 Mc-Farland standard) at solidified Macconkey agar and mannitol salt agar were incubated at 37 °C after preparing well of 6 mm diameter using sterile cork borer. Different concentrations of synthesized nanostructures (500 and 1000 μg/50 μl) were applied as low and high dose compared with ciprofloxacin (5 μg/50 μl) and DIW (50 μl) as control positive and negative, respectively. The overnight incubated Petri dishes at 37 °C declared inhibition zones (mm) which were measured using vernier caliper. The antibacterial activity measured in terms of inhibition zones (mm) was declared statistically significant using one-way analysis of variance (ANOVA) with SPSS 20.0.

### Molecular Docking Studies

Cell wall synthesis has been considered an effective target for discovery of various antibiotics having different mode of actions, like beta-lactam and glycopeptide antibiotics. Beta-lactam antibiotics have been reported as the most highly marketed drugs and represents most common treatment for bacterial infections [39, 40]. They inhibit cell wall biosynthesis by binding with penicillin-binding protein (PBP) and *β*-lactamases [41]. Similarly, enzymes (i.e., DNA gyrase) belonging to nucleic acid synthesis have also been considered as effective target for antibiotics discovery [42]. Here, we performed molecular docking studies of SnO_2_ NPs against *β*-lactamase and DNA gyrase enzyme of both *E. coli* and *S. aureus* to have an insight into their possible mode of action.

**Fig. 4:**
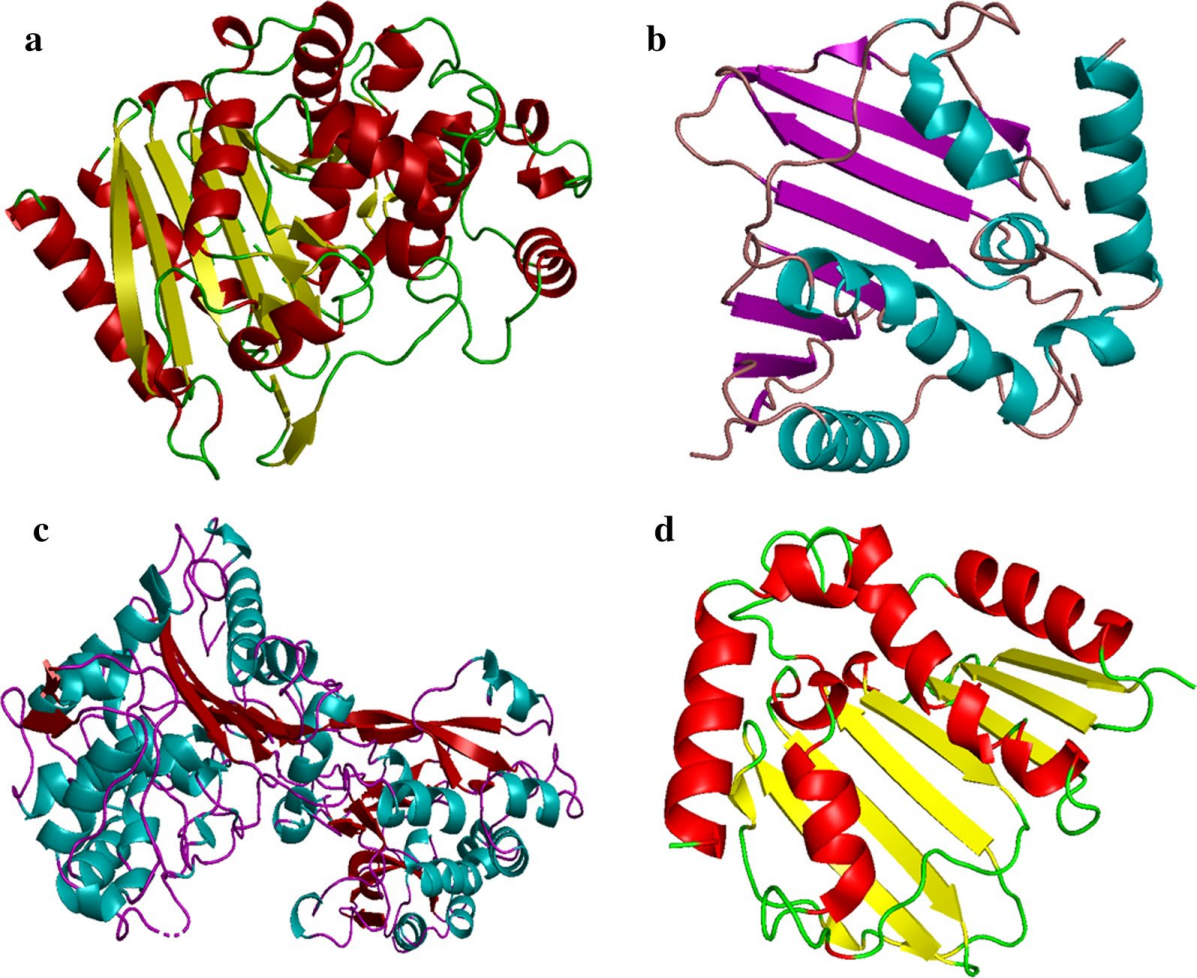
3D-structure of **a** beta lactamase (PDB: 4KZ9), **b** DNA gyrase (PDB: 6KZX) from *E. coli*, **c** beta lactamase (PDB: 1MWU) and **d** DNA gyrase (PDB: 5CTU) from *S. aureus*

The 3D-crystal structures of selected protein targets belonging to *E. coli* and *S. aureus* were obtained from protein data bank with accession code: 4KZ9; Res: 1.72 Å [43], 1MWU; Res: 2.6 Å [44] for *β*-lactamase, while 6KZX; Res: 2.1 Å [45] and 5CTU; Res: 1.45 Å [46] for DNA gyrase as shown in Fig. 4.

Molecular docking studies were carried out using ICM Molsoft software (Molsoft L.L.C., La Jolla, CA) software [47]. Receptor preparation tool of ICM was utilized for protein structure involving addition of polar hydrogen atoms, removal of water molecules and co-crystallized ligand. Protein structures were optimized using energy minimization tool with default parameters while grid box was used to identify active pocket around crystallized ligand. Later, 10 best conformations specifying orientation of SnO_2_ NPs inside active pocket of enzyme were generated in each case. Lowest binding scored conformation was selected for further analysis that unveiled binding pattern and inhibition tendency of fabricated NPs against these selected enzymes.

The SnO_2_ structure was generated using ligedit tool of ICM while 3D view of docked conformation was generated through ICM and discovery studio visualizer [48].

### Materials Characterization

BRUKER D2 Phaser x-ray diffraction (XRD) ranged 2*θ* = 10°–70° with Cu Kα (*λ* = 1.540 Å) was used to examine lattice structure and retrieve data about phase constitution. Functional groups of the synthesized products were evaluated through PerkinElmer Fourier-transform infrared (FTIR) spectrometer. JEOL JSM-6610LV field emission scanning electron microscope (FESEM) along with Oxford XMax EDS detector with INCA software was employed to analyze morphology and elemental composition of prepared samples. Philips CM30 along with JEOL JEM 2100F high-resolution transmission electron microscope (HR-TEM) was employed to record SAED, HRTEM images and lattice fringe patterns. The optical properties of prepared tin oxide samples were recorded from 300 to 800 nm via GENESYS-10S UV–vis spectrometer.

## Result and discussion

**Fig. 5:**
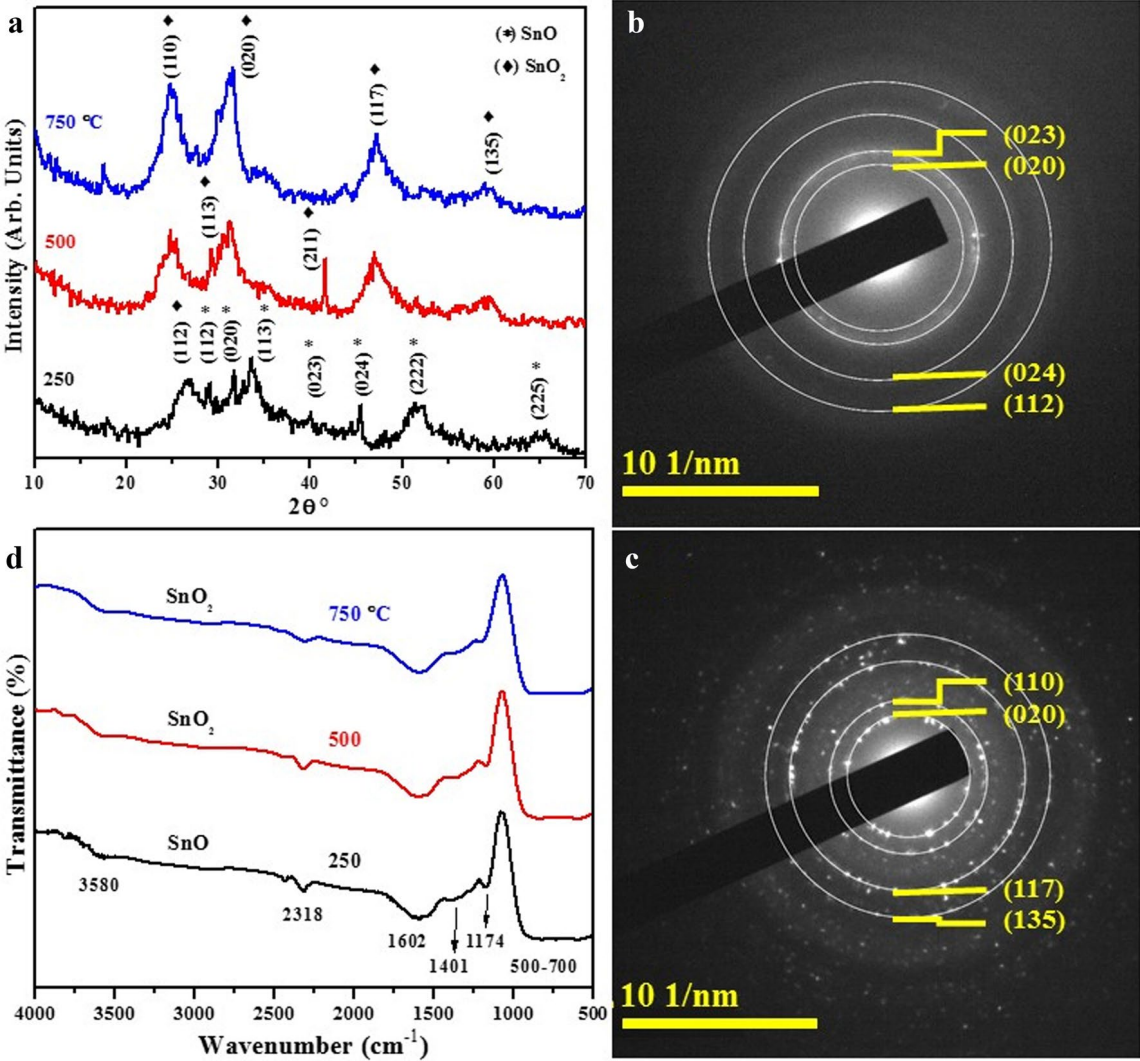
**a** XRD spectra obtained from SnO annealed at 250, 500 and 750 °C **b**, **c** SAED rings of 250 and 750 °C samples, respectively, and **d** FTIR patterns

Figure 5(a) presents XRD spectra obtained from tin oxide samples synthesized and annealed at various temperatures. For phase compositional analysis, CuKα radiation was employed while Debye–Scherer relation, $D = K\lambda /\beta {\mathrm{Cos}}\theta$ where *λ* = 1.54 Å and *k* = 0.9 was used to calculate crystallite size of the material. At 250 °C, XRD pattern shows peaks with 2θ values of 29.12° (112), 31.60° (020), 33.57° (113), 40.15° (023), 45.54° (024), 51.35° (222) and 64.57° (225) which are all ascribed to SnO orthorhombic phase (JCPDS: 01–077-2296). Only one peak recorded at 26.66° (112) belongs to SnO_2_ orthorhombic structure (JCPDS: 01–078-1063). As samples were annealed at 500 and 750 °C, the diffraction peaks related to SnO vanished and the resulting product was identified as orthorhombic SnO_2_ with crystallographic planes 24.81° (110), 29.12° (113), 31.23° (020), 41.59° (211), 46.90° (117), 59.57° (135) that well matched with JCPDS file No. 01-078-1063 [49]. The observed XRD results indicate that the prepared material was first oxidized to SnO at 250 °C. Later, at and above 500 °C, it fully transformed to SnO_2_ [50]. The results also show that crystallinity of samples enhanced with increasing temperature. Furthermore, SAED patterns obtained from samples annealed at 250 and 750 °C depicted bright spot rings as illustrated in Fig. 5b, c, respectively. The analyzed patterns with lattice planes (020), (023), (024) and (112) are assigned to orthorhombic SnO (Fig. 5b) and (020), (110), (117) and (135) diffraction planes are attributed to SnO_2_ nanomaterial (Fig. 5c) for samples annealed at 250 and 750 °C, respectively. Crystal nature of products was also confirmed via SAED images which is consistent with observations of XRD.

FTIR spectra of fabricated tin oxide nanomaterials heated at 250, 500 and 750 °C are presented in Fig. 5d. The absorption peak centered at about 3580 cm^−1^ coupled with almost 1602 cm^−1^ band was attributed to stretching/bending of O–H group and Sn-OH bond due to the fact that tin oxide absorbs certain amount of water from ambient atmosphere [51]. Low absorption peak ranged from 2300 to 2400 cm^−1^ is assigned to carbon dioxide which was created in products upon exposure to the atmosphere [52]. The bonds appearing at 1174 cm^−1^ correspond to vibrations of various kinds of surface hydroxyl groups while peak at 1401 cm^−1^ is due to C–H bending vibrations [53, 54]. The characteristic peaks ranged at 500–700 cm^−1^ are attributed to surface layer Sn–O vibrations [55]. An increase in the annealed temperature causes blueshift in peaks while the characteristic peaks appear strong and more intense. This may be due to the annealing process where atoms of nanomaterial acquire enough energy to change position of nanoparticle atoms resulting in recrystallization [56].

**Fig. 6:**
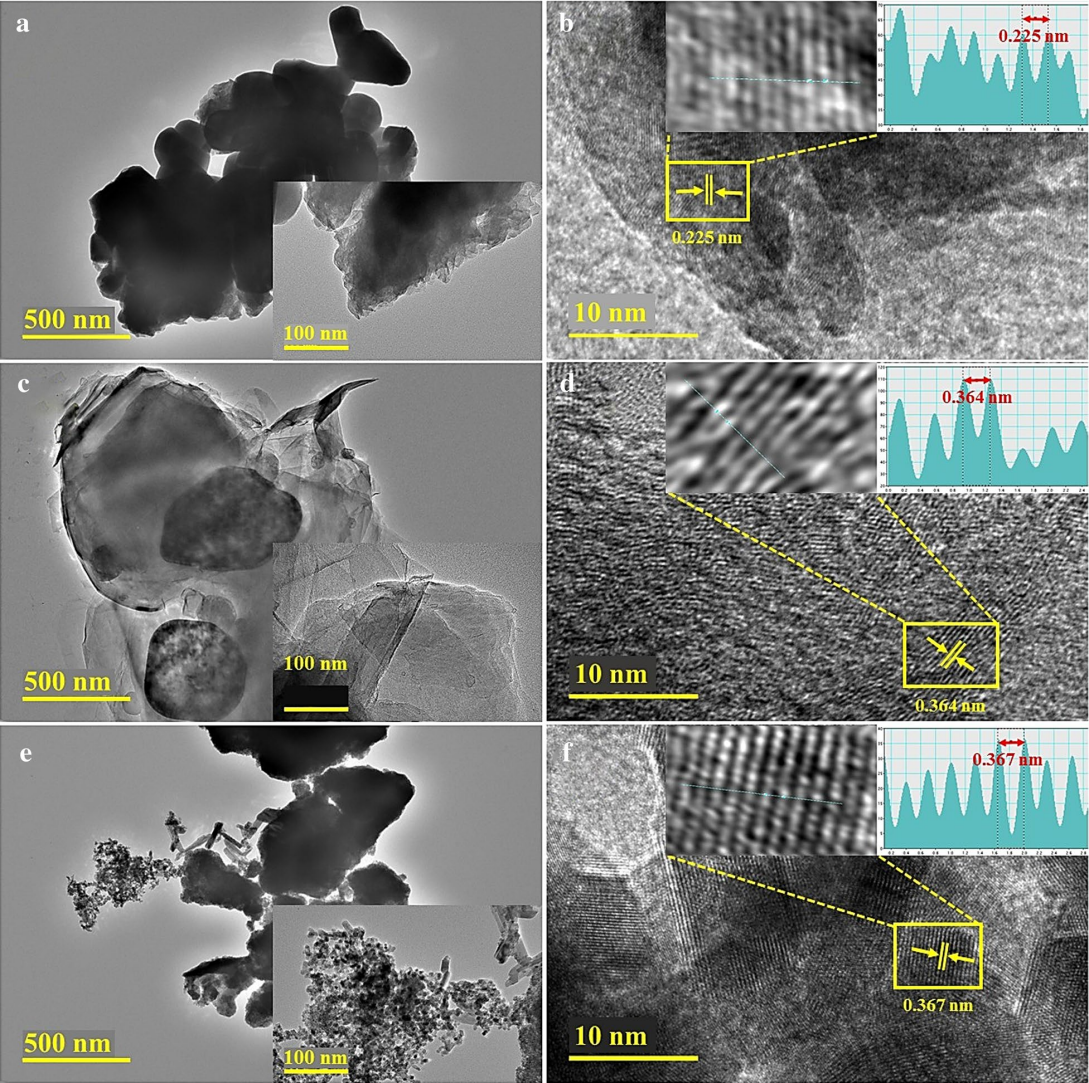
**a**–**c** HR-TEM and **d**–**f** lattice fringes of nanostructures obtained at annealing temperatures of 250, 500 and 750 °C, respectively

FESEM and HRTEM analysis was carried out to collect detailed information of morphology and nanostructure of fabricated products. HRTEM images of tin oxide annealed at 250, 500 and 750 °C were recorded, as depicted in Fig. 6a–c. The images provide evidence for the formation of agglomeration within nanoparticles with nearly spherical shape and random distribution of particle size (see Fig. 6a, c). In Fig. 6b, large size, slightly transparent and monodisperse particles with little agglomeration could be observed. Same behavior of particles distribution can be observed from inset images at high magnification. The sharp change in morphological behavior at 500 °C is noticeable. This temperature may be a characteristic feature during the oxidation process [22]. Further, Fig. 6d–f displays HRTEM micrographs obtained from sample annealed at 250, 500 and 750 °C, respectively, to detect interplanar distance. In crystallites, spacing of lattice fringes is ~ 0.225 nm as depicted in inset IFFT profile image in Fig. 6d which corresponds to (023) diffraction plane of orthorhombic phase of SnO (JCPDS: 01-077-2296). The d-spacing calculated from lattice fringes in samples treated at 500 and 750 °C is about 0.364 and 0.367 nm, which are attributed to (110) plane of SnO_2_ orthorhombic structure according to JCPDS NO. 01-078-1063. These results are in good agreement with the XRD findings [49, 57, 58]. Provided IFFT images clearly show increase in d-spacing with increasing temperature.

**Fig. 7:**
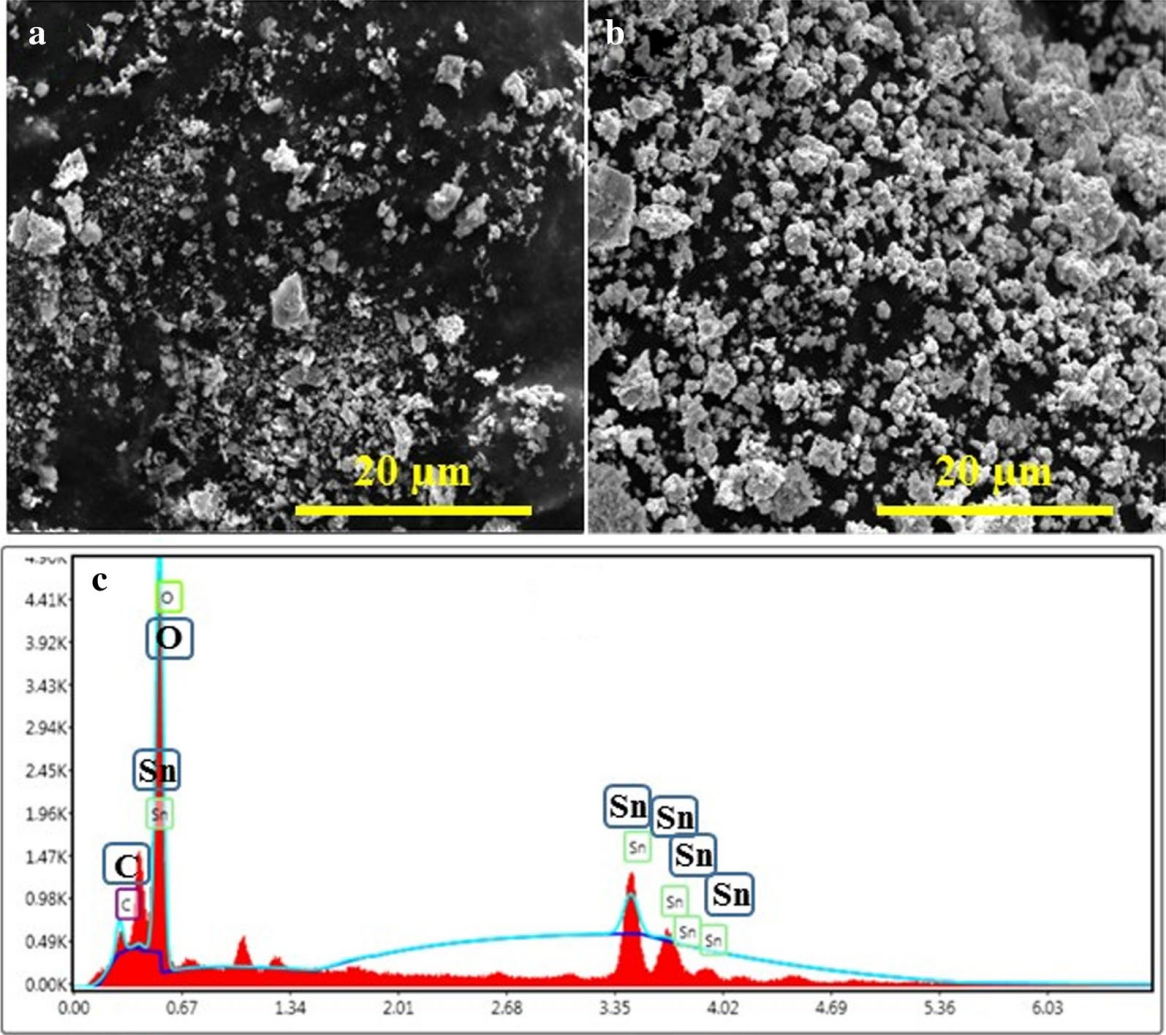
FESEM micrographs from samples annealed at **a** 250 and **b** 750 °C and **c** EDX spectrum from sample annealed at 750 °C

FESEM images of tin oxide annealed at 250 (Fig. 7a) and 750 °C (Fig. 7b) depict variation in shape and size of lattice structures such as non-uniform or random shape of particles with large and small individual grains along with agglomeration. Figure 7b shows FESEM micrograph of the sample annealed at 750 °C which signifies small agglomerated particles and a well-grown structure in comparison with the sample annealed at low temperature with random distribution of particles. Agglomeration of particles results in a reduction of surface free energy due to an increase in their size resulting in a decrease in their surface area. Agglomeration of nanoparticles is due to adhesion of particles to each other by weak forces leading to (sub) micron-sized entities. [59].

The composition of fabricated tin oxide annealed at 750 °C was analyzed with EDX technique as depicted in Fig. 7c. Sample spectra clearly exhibited the presence of Sn, O and C with weight % of 53.7, 42.2 and 4.0%, respectively. No impurity constituents were present in the product which suggested high purity of SnO_2_ nanomaterial while C content may originate from carbon tabs used to hold samples.

**Fig. 8:**
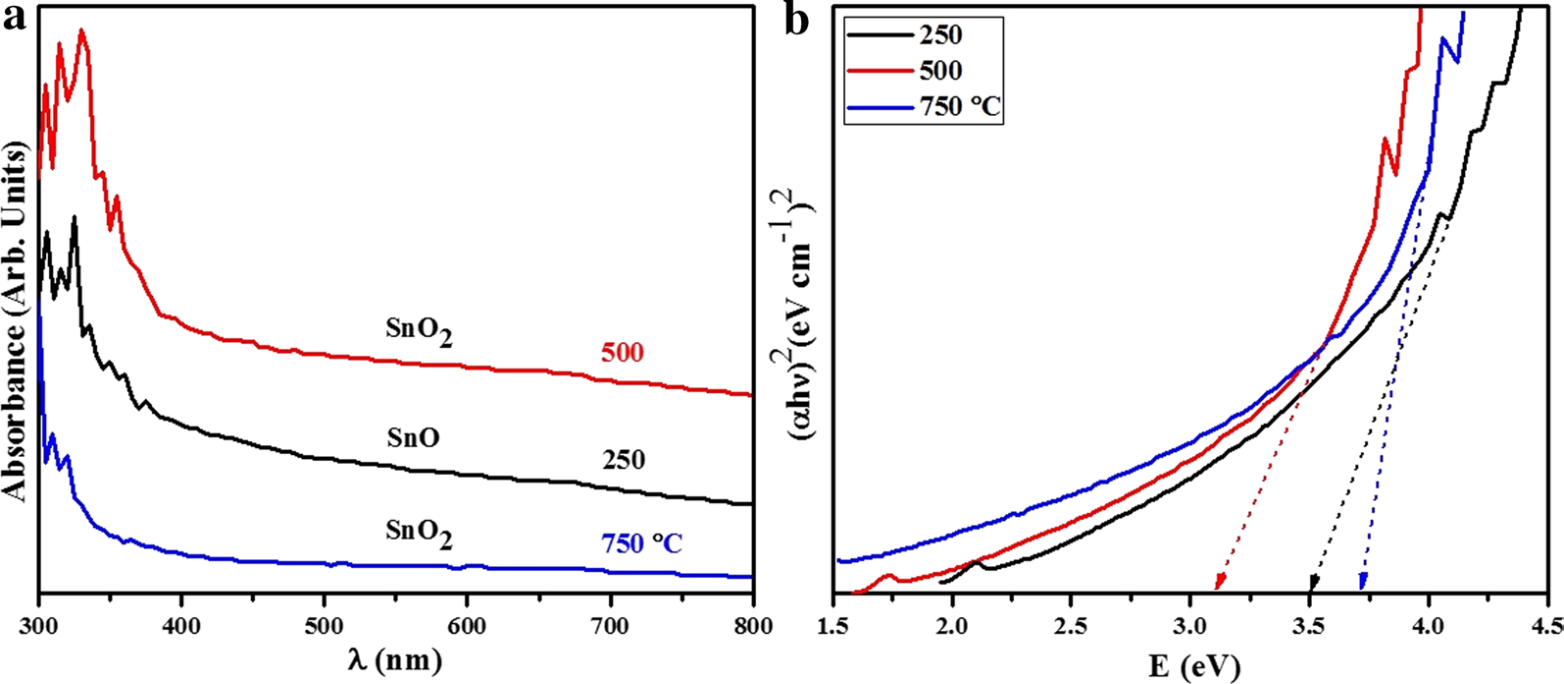
**a** UV–Vis absorbance spectra and **b** energy gap values obtained from samples annealed at 250, 500 and 750 °C, respectively

A non-destructive absorption spectroscopy technique was used to study optical properties of conducting and semiconducting nanomaterials. Absorption spectra obtained from tin oxide annealed at various temperatures are illustrated in Fig. 8a. Several factors affect expected absorbance such as oxygen deficiency, energy band gap, impurity nature and surface roughness. Spectra obtained from annealed tin oxide exhibited ultra-violet cut-off at 300–365 nm [60], which may be due to photo-excitation of electrons from valance to conduction band. The absorption spectra decreased slightly between 300 and 365 nm with increasing temperature as shown in Fig. 8a. To calculate band gap, Tauc relation $\alpha h\nu =A {(h\nu -{E}_{\mathrm{g}})}^{n}$ where *α* stands for absorption coefficient, *A* represents constant while *n* = 1/2 for direct band gap. An extrapolation of ${(\alpha h\nu )}^{2}$ versus hν plot provides optical band gap value *E*_g_ (Fig. 8b). Measured band energies were 3.51, 3.32 and 3.71 eV for samples annealed at 250, 500 and 750 °C, respectively [61, 62]. The band gap of products is consistent with reported values in the literature [61]. Overall, as temperature increases, the atoms of nanoparticles attain more energy to change their position for recrystallization which alter their morphology and reduce grain size (can be analyzed from HRTEM data). As particle size decreases, band gap value increases which is attributed to normal quantum confinement effect. A similar trend was observed by Malik et al. [63]. The lowest band gap exhibited by nanomaterial annealed at 500 °C was ascribed to unique nanostructures or amorphous synthetization of polycrystalline tin oxide and generation of oxygen vacancies that produce redshift. Tauc model states that amorphous or disordered surface semiconductors have localized band tail states with lower band energy [64].

**Fig. 9:**
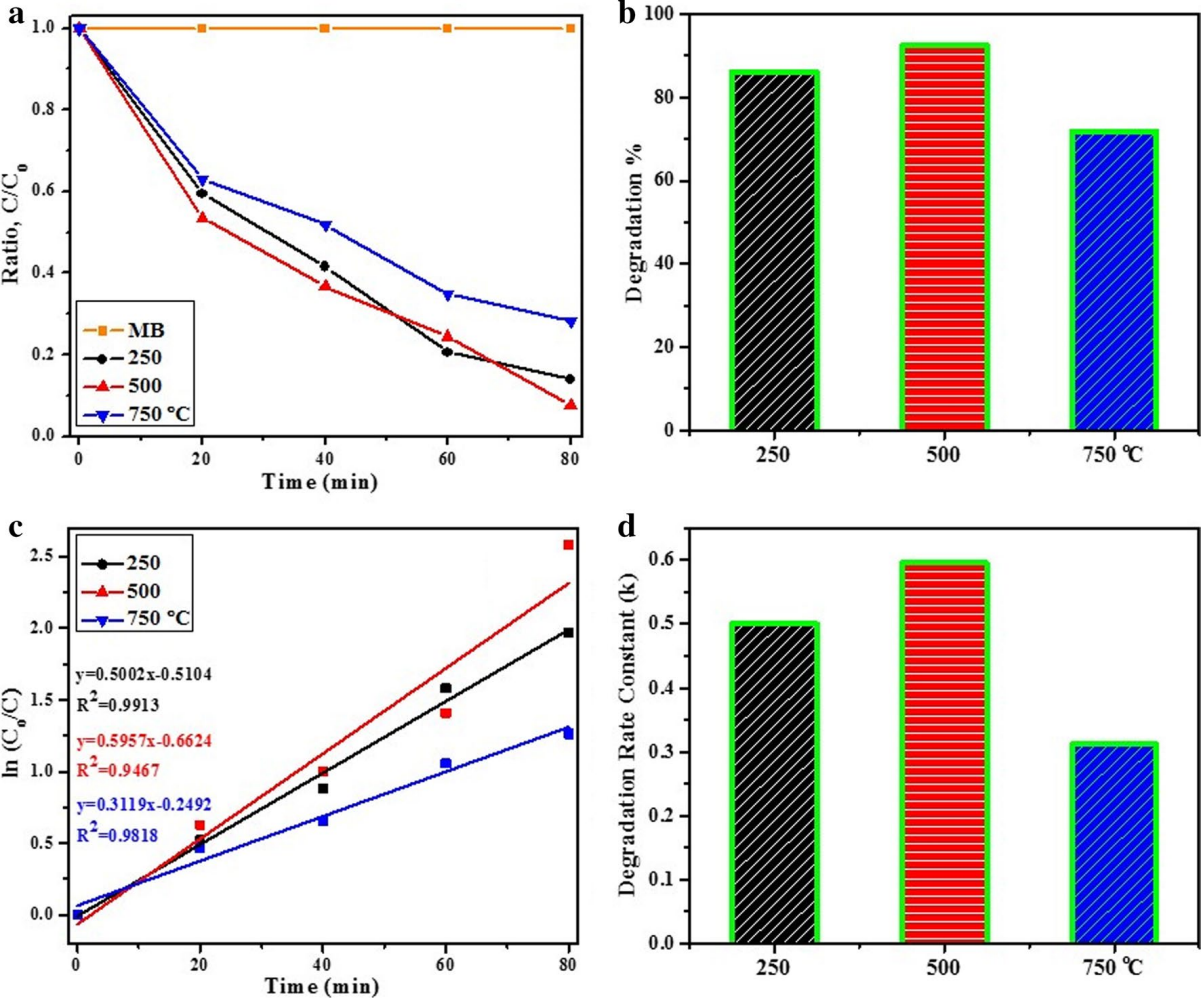
**a** Results of MB degradation exhibited by fabricated photocatalysts **b** degradation % bar graph **c** ln (C_o_/C) vs. irradiation time plot and **d** calculated degradation rate constant graph

The photoactivity of tin oxide products annealed at various temperatures was examined by undertaking photodegradation of MB dye under light irradiation (Fig. 9). Variation in MB solution optical absorption at *λ*_max_ = 665 nm during its photo-decomposition is depicted in Fig. 9a. Addition of fabricated samples produces a decrease in MB absorption band with time. The maximum photocatalytic performance was exhibited by the sample annealed at 500 °C (*E*_g_ = 3.32 eV) which is attributed to specific morphology and low electron–hole recombination rate. We observed 86.0, 92.4 and 71.6% MB degradation by tin oxide photocatalysts prepared by annealing at 250, 500 and 750 °C in 80 min, respectively (Fig. 9b). The photoactivity of semiconductor materials is also related to their band gap energy which influences the redox potential of photogenerated electron–hole pair during MB degradation process. Among three samples tested here, photocatalyst annealed at 500 °C presented the lowest band gap energy (3.32 eV) while considerably enhanced degradation % exhibited by this product was credited to its unique structure and high degree of agglomeration as depicted in Fig. 9b. A pseudo-first-order dye degradation is illustrated using ln (*C*_o_/*C*) vs. irradiation time plot: ln (*C*_o_/*C*) = *kt* as exhibited in Fig. 9c where *k* denotes rate constant, *C*_o_ and C stands for initial and final concentration of dye (MB), respectively [63, 65–67]. The value of k using nanomaterial prepared at 500 °C was 0.59 min^−1^ and significant decrease in samples synthesized at 250 and 750 °C was observed at about 0.50 and 0.31 min^−1^, respectively (Fig. 9d).

**Fig. 10:**
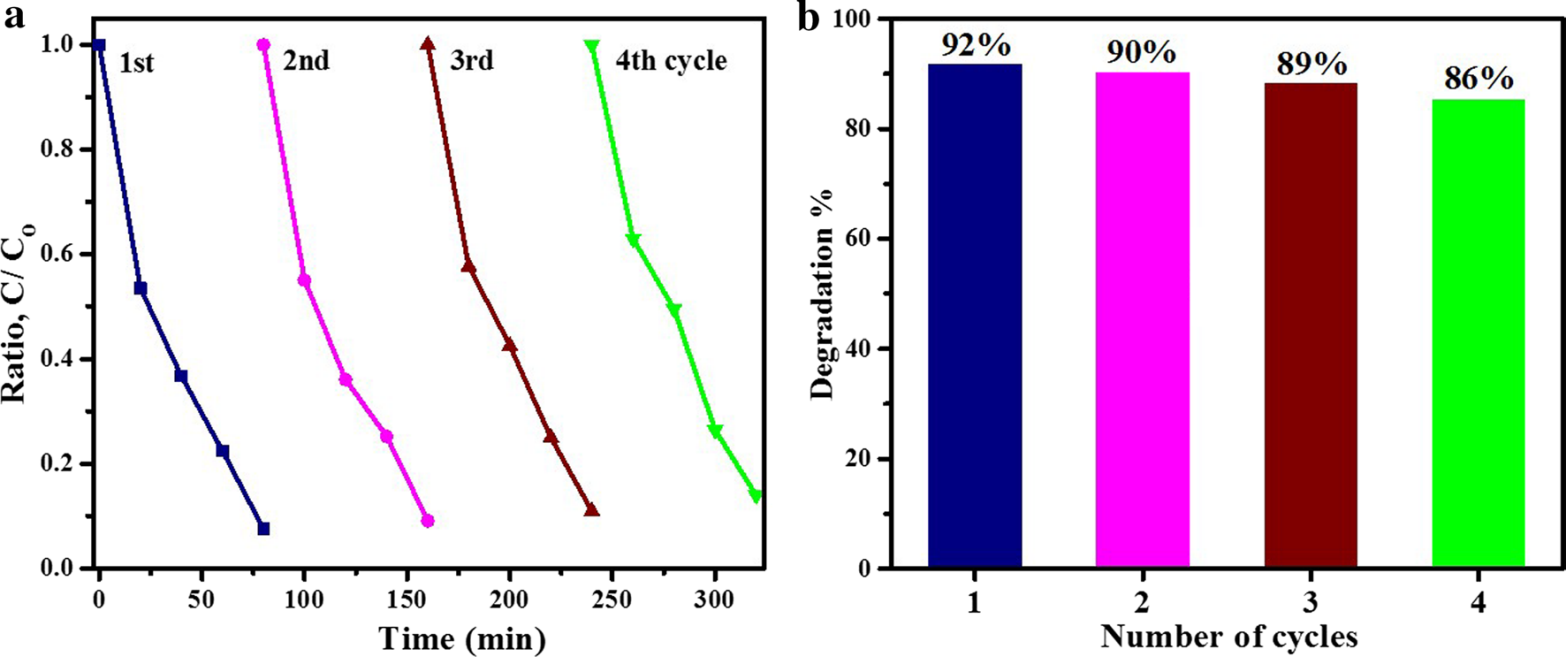
**a** Reusability of tin oxide photocatalyst annealed at 500 °C and **b** degradation % bar graph

For treatment of polluted water, photocatalysts require to exhibit stability and reusability for long periods of time to make the process economically feasible. In the present study, four cycles of tests were performed to remove MB using fabricated tin oxide photocatalysts to determine their stability. The observation of four consecutive cycles of dye degradation are presented in Fig. 10a, b. Nanomaterial annealed at 500 °C depicted a slight decrease in MB photo-decomposition after four cycles (6% decrease). The results of contaminant removal from water reported in this study are comparable with those reported by Prakash et al. [65].

**Fig. 11:**
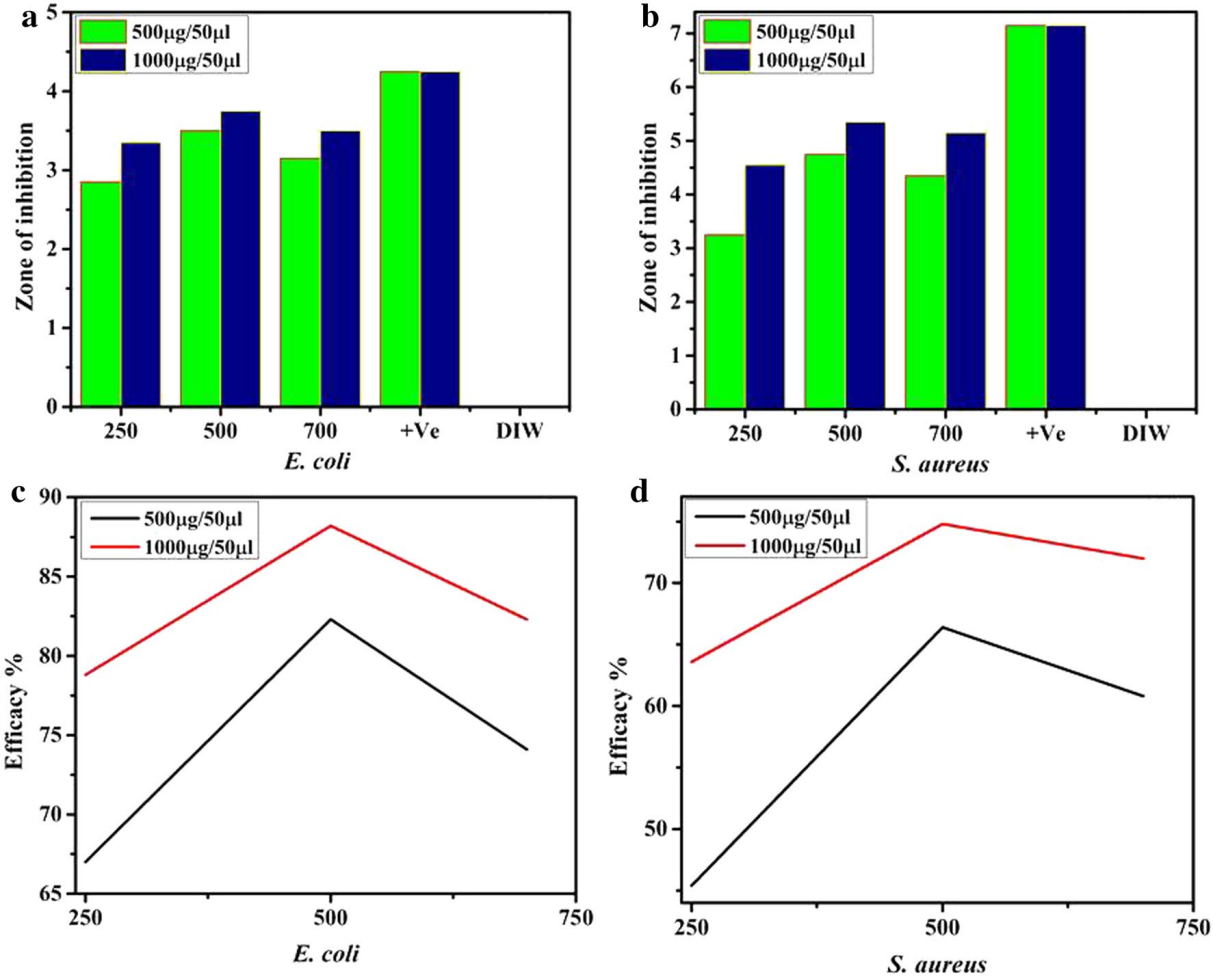
**a** In vitro bactericidal action of SnO annealed at different temperatures for *E. coli* **b** *S. aureus* **c** In vitro bactericidal efficacy %age of fabricated NPs for *E. coli* **(d)** and *S. aureus*, respectively

SnO nanostructures fabricated at various temperature treatments in vitro antibacterial activity measured in terms of inhibition zones (mm) along with comparative efficacy %age are shown in Fig. 11a–d and Table 1. The graphs present direct proportion between nanostructures concentration and zones of inhibition formed. Significant zones of inhibition recorded for SnO (250, 500 and 750 °C) samples for *E. coli* and *S. aureus* ranged between 2.85–3.5 mm, 3.35–3.75 mm and 3.25–4.75 mm and 4.55–5.35 mm at low and high concentrations, respectively, Fig. 11a, b and Table 1. The efficacy %age of synthesized nanomaterials increased from 67.0–82.3 to 78.8–88.2% for *E. coli* and similarly, 45.4–66.4% and 63.6–74.8% for *S. aureus*, respectively, Fig. 11c, d. All measured results were compared with DIW (0 mm). Positive control depicted 4.25 mm and 7.15 mm inhibition zones for *E. coli* and *S. aureus*, respectively, Fig. 11a, b. Overall SnO_2_ nanostructures optimized at 500 °C found more potent at both concentrations and more broadly, SnO_2_ found more potent against gram-negative (G –ve) *E. coli* compared with gram-positive (G +ve) *S. aureus*.

**Table 1:** Bactericidal action of prepared samples

Sample	Inhibition zone (mm)^a^		Inhibition zone (mm)^b^	
	500 μg/50 μl	1000 μg/50 μl	500 μg/50 μl	1000 μg/50 μl
250 °C	2.85	3.35	3.25	4.55
500 °C	3.5	3.75	4.75	5.35
750 °C	3.15	3.5	4.35	5.15
Ciprofloxacin	4.25	4.25	7.15	7.15
DIW	0	0	0	0

^a^Zones of inhibition (mm) of fabricated products for *E. coli*

^b^Attained nanostructures inhibition zones for *S. aureus*

Size, concentration and morphology of nanostructures directly affects oxidative stress produced. Antibacterial activity imperiling size and concentration portrays inverse relation to size [68–70]. Nanostructures more efficiently produce reactive oxygen species (ROS) which exist in bacterial membrane resulting cellular organelles extrusion and bacteria death [71]. SnO_2_ generate more efficiently ROS including hydrogen peroxide (H_2_O_2_), OH groups and superoxide ions [72]. The increased antibacterial efficacy of fabricated SnO at various temperature treatments for *E. coli* compared to *S. aureus* could be attributed to difference in cell wall structures of bacteria. G –ve bacteria cell wall consists of peptidoglycan thin layer with an outer membrane containing proteins and phospholipids while G  +ve cell wall contains thick layer of peptidoglycan with lipoteichoic and teichoic acids. This major difference in cell wall structure of both bacteria leads toward maximum efficacy of fabricated nanostructures toward G −ve compared to G  +ve bacteria [18].

Resistance acquired by microbial pathogens against various antibiotic drugs especially multidrug resistance pose huge threat to public health around the globe and there is an urgent need of more antibiotic drugs with novel mode of action [73]. Antibiotics belonging to various classes follow different mechanisms for their activity and target pathways vital for bacterial survival. For instance, Beta-lactam antibiotics such as penicillin target enzymes involved in peptidoglycan synthesis (i.e., important precursor for cell wall synthesis) [74] while Rifampicin a well-known antibiotic target enzyme belongs to nucleic acid biosynthetic pathways [42] suggesting importance of both cell wall and nucleic acid biosynthetic pathways as target for new antibiotic discovery [75]. Although bactericidal activity of various nanoparticles has been reported previously in recent years still exact mechanism of their action is not known [76, 77]. Keeping in view good antibacterial activity of SnO_2_ against *E. coli* and *S. aureus*, we performed molecular docking studies to identify their possible mechanism of action against *β*-lactamase and DNA gyrase enzymes as potential target.

**Fig. 12:**
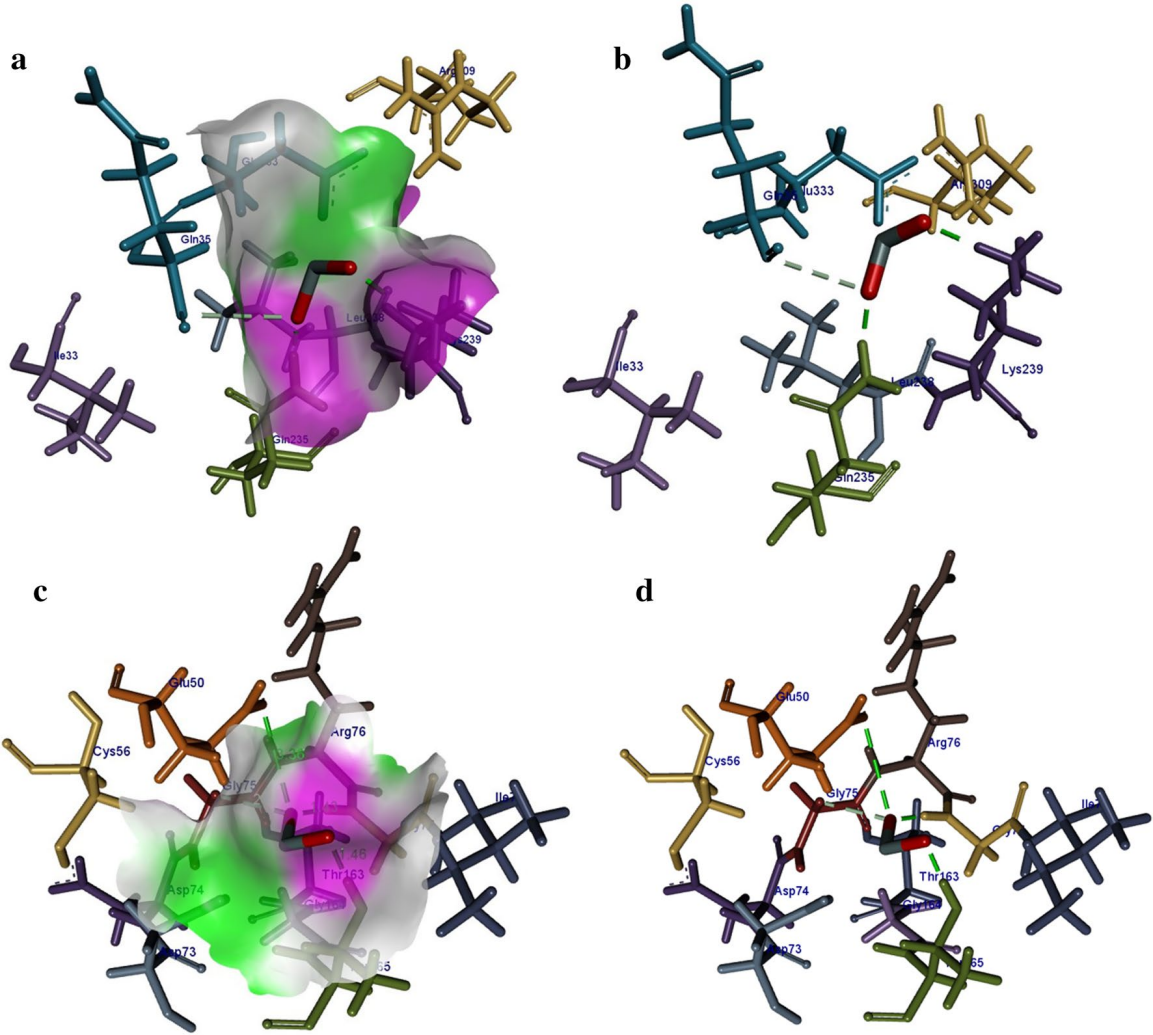
**a**, **b** Binding interaction pattern of SnO_2_ nanoparticle with active site residues of *β*-lactamase and **c**, **d** DNA gyrase from *E. coli*

In case of *β*-lactamase from *E. coli* the best binding score observed was − 5.71 kcal/mol showing H-bonding interaction with Lys239 (1.80 Å) and Gly235 (1.66 Å) alongside metal contact interaction with Gln35 as shown in Fig. 12a, b. Similarly, the top binding score obtained for DNA gyrase from *E. coli* was − 9.57 kcal/mol having H-bonding interaction with Thr163 (1.46 Å), Gly77 (1.43 Å) and Glu50 (3.36 Å) along with metal contact interaction with Gly75 as depicted in Fig. 12c, d.

**Fig. 13:**
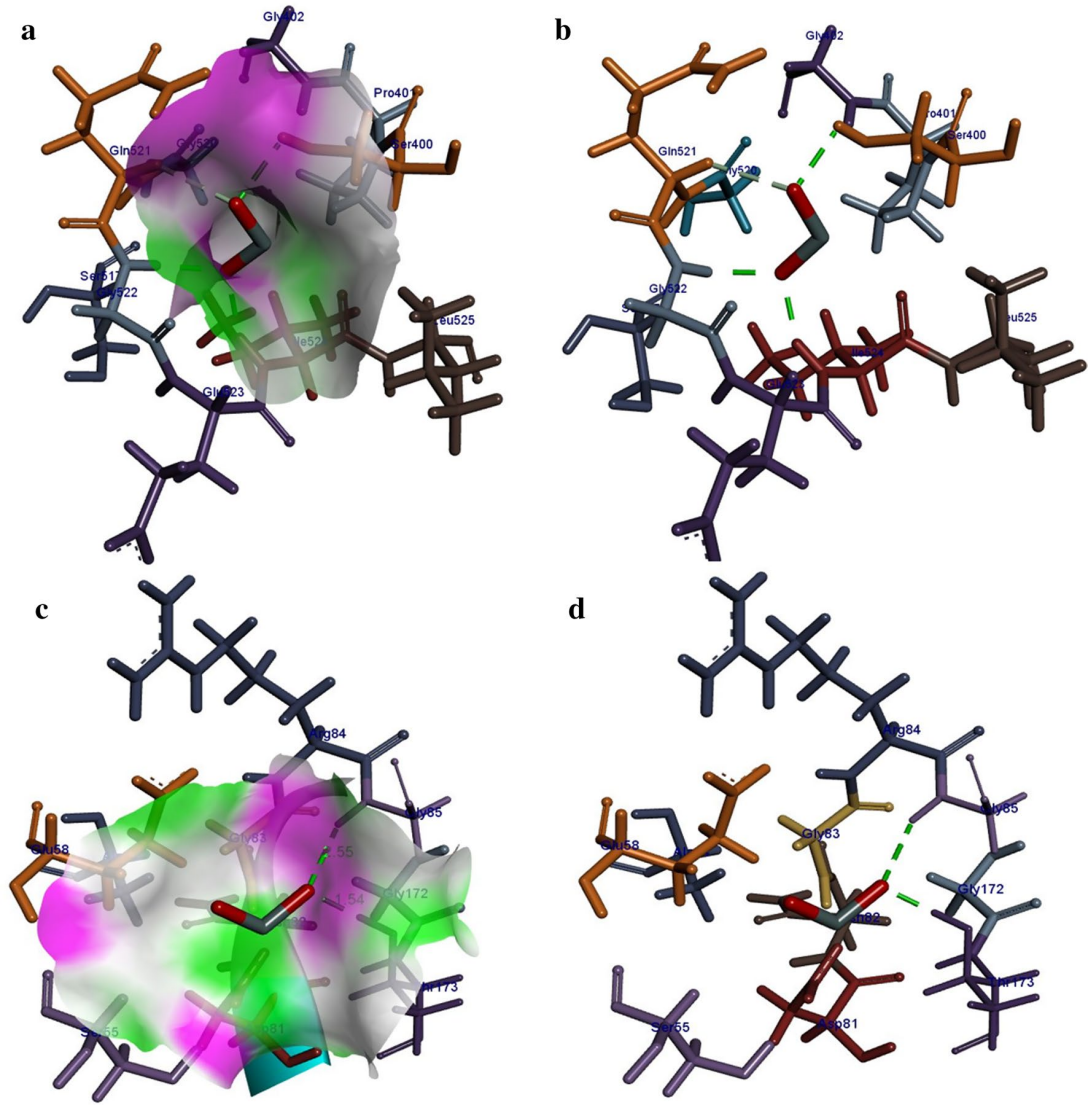
**a**, **b** Binding interaction pattern of SnO_2_ nanoparticle with active site residues of *β*-lactamase and **c**, **d** DNA gyrase from *S. aureus*

The best binding score obtained for *β*-lactamase of *S. aureus* was − 11.83 kcal/mol. The binding patterns of SnO_2_ inside active pocket involved H-bonding interaction with Ser400 (2.16 Å), Gly522 (1.99 Å) and Ileu524 (1.90 Å). In addition, metal contact interaction was observed between SnO_2_ and Gln521 as depicted in Fig. 13a, b. For DNA gyrase from *S. aureus* the best conformation obtained showed H-bonding interaction with Gly85 (2.55 Å) and Thr173 (1.54 Å) having binding score − 8.61 kcal/mol (Fig. 13c, d).

## Conclusion

In summary, tin oxide nanoparticles annealed at various temperatures were synthesized by a facile and simple precipitation process. Annealing of samples at 250 °C during synthesis produced predominantly orthorhombic SnO which transformed to SnO_2_ at 500 and 750 °C. The XRD and HRTEM analyzes revealed that nanoparticles possessed orthorhombic structure while particle size increased initially with an increase in temperature to 500 °C followed by a decrease at 750 °C. Large particle size at 500 °C was attributed to unique structure exhibited by the sample annealed at that temperature. Fabricated nanostructures demonstrated well-crystallized behavior along with agglomeration. Photoactivity of annealed tin oxide nanomaterials was evaluated by decomposing MB dye that was used as a model organic contaminant and a comparison between annealed samples was realized. Product synthesized at 500 °C exhibited 6% higher activity compared to sample annealed at 250 °C during degradation of MB. Maximum MB degradation attained in this study was 92% after 80 min irradiation time by 500 °C sample. It is suggested that radical (*OH^1−^ and *O_2_^−^) and holes are major active entities in photocatalysis process. In silico predictions are in good agreement with in vitro bactericidal activity of SnO_2_ NPs. Molecular docking studies of SnO_2_ NPs against selected enzymes, i.e., beta lactamase and DNA gyrase, suggested their tendency to impede activity of these enzymes that need to be further explored and confirmed through enzyme inhibition assay studies.

## Data Availability

All data are fully available without restriction.

## Abbreviations

EDS: Energy-dispersive x-ray spectroscopy
FTIR: Fourier-transform infrared spectroscopy
G +ve: Gram-positive
G −ve: Gram-negative
HR-TEM: High-resolution transmission electron microscopy
JCPDS: Joint Committee on Powder Diffraction Standards
NPs: Nanoparticles
SnO: Tin oxide
UV–Vis: Ultra-violet visible spectroscopy
XRD: X-ray diffraction
